# No Evidence of Neandertal mtDNA Contribution to Early Modern Humans

**DOI:** 10.1371/journal.pbio.0020057

**Published:** 2004-03-16

**Authors:** David Serre, André Langaney, Mario Chech, Maria Teschler-Nicola, Maja Paunovic, Philippe Mennecier, Michael Hofreiter, Göran Possnert, Svante Pääbo

**Affiliations:** **1**Max Planck Institute for Evolutionary AnthropologyLeipzigGermany; **2**Laboratoire d'Anthropologie Biologique, Musée de l'HommeParisFrance; **3**Laboratoire de Génétique et Biométrie, Université de GenèveGenèveSwitzerland; **4**Department of Anthropology, Natural History MuseumViennaAustria; **5**Institute of Quaternary Paleontology and Geology, Croatian Academy of Sciences and ArtsZagrebCroatia; **6**Ångström Laboratory, Uppsala UniversityUppsalaSweden

## Abstract

The retrieval of mitochondrial DNA (mtDNA) sequences from four Neandertal fossils from Germany, Russia, and Croatia has demonstrated that these individuals carried closely related mtDNAs that are not found among current humans. However, these results do not definitively resolve the question of a possible Neandertal contribution to the gene pool of modern humans since such a contribution might have been erased by genetic drift or by the continuous influx of modern human DNA into the Neandertal gene pool. A further concern is that if some Neandertals carried mtDNA sequences similar to contemporaneous humans, such sequences may be erroneously regarded as modern contaminations when retrieved from fossils. Here we address these issues by the analysis of 24 Neandertal and 40 early modern human remains. The biomolecular preservation of four Neandertals and of five early modern humans was good enough to suggest the preservation of DNA. All four Neandertals yielded mtDNA sequences similar to those previously determined from Neandertal individuals, whereas none of the five early modern humans contained such mtDNA sequences. In combination with current mtDNA data, this excludes any large genetic contribution by Neandertals to early modern humans, but does not rule out the possibility of a smaller contribution.

## Abstract 2

Analysis of mitochondrial DNA from four Neandertal fossils and five "modern human" contemporaries excludes any large genetic contribution of Neandertals to the gene pool of modern humans

## Introduction

Despite intense research efforts, no consensus has been reached about the genetic relationship between early modern humans and archaic human forms such as the Neandertals. While supporters of “multiregional evolution” argue for genetic exchange or even continuity between archaic and modern humans (Weidenreich 1943; Wolpoff et al. 1984, Wolpoff et al. 2000; Duarte et al. 1999; Hawks and Wolpoff 2001), proponents of a “single African origin” of contemporary humans claim that negligible genetic interaction took place (Cann et al. 1987; Stringer and Andrews 1988; Ingman et al. 2000; Underhill et al. 2000; Stringer 2002). Mitochondrial DNA (mtDNA) sequences from early modern humans would in principle be able to resolve the question of a contribution of Neandertal mtDNA to modern humans. However, human DNA is pervasive in palaeontological and archaeological remains as well as in most laboratory environments (e.g., Krings et al. 2000; Hofreiter et al. 2001b; Wandeler et al. 2003). It is therefore currently impossible to differentiate contaminating modern DNA sequences from endogenous human DNA in human remains. Thus, although mtDNA sequences have been reported from remains of early modern humans (Adcock et al. 2001; Caramelli et al. 2003), it is not possible to determine whether such DNA sequences indeed represent endogenous DNA sequences (Abbott 2003). A related problem is that if a Neandertal fossil yields modern human-like DNA sequences, those might be discarded as putative contaminations (Nordborg 1998; Trinkaus 2001), even if they may be endogenous and represent evidence for a close genetic relationship or interbreeding between the two groups.

To explore the genetic relationship between early modern humans and Neandertals in spite of these difficulties, we made use of the fact that the four Neandertal mtDNA sequences determined to date can easily be distinguished from those of modern humans (Krings et al. 1997, Krings et al. 2000; Ovchinnikov et al. 2000; Schmitz et al. 2002; Knight 2003). This allowed us to ask whether all well-preserved Neandertal remains contain Neandertal-like mtDNA and whether all well-preserved early modern human remains fail to contain such DNA sequences. Thus, we did not attempt to determine DNA sequences that are similar to present-day human mtDNA. Instead, we determined whether Neandertal-like mtDNA sequences were present or absent in well-preserved remains of Neandertals and of early modern humans.

## Results and Discussion

**Figure 1:**
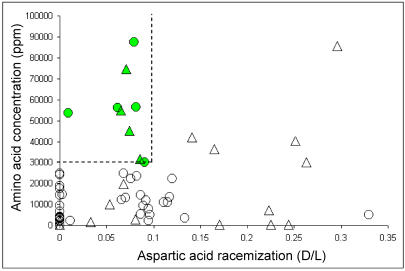
Amino Acid Analyses of 64 Hominid Remains For each bone, the extent of aspartic acid racemization (D/L) and the amino acid concentration (ppm) is given. The dash lines delimit the area of amino acid preservation compatible with DNA retrieval. Circles and triangles represent early modern humans and Neandertals, respectively. The samples from which DNA extractions were performed are green (see also Table S1).

**Figure 2:**
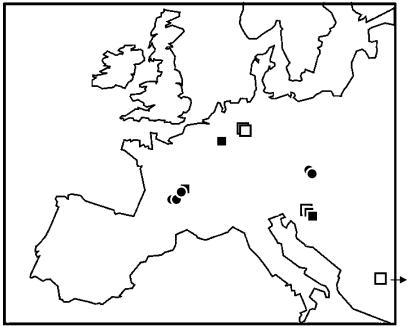
Geographical Origin of Neandertal and Early Modern Human Samples from Which mtDNA Sequences Have Been Analyzed Filled squares and filled circles represent Neandertal and early modern human remains, respectively, analyzed in this study. The four Neandertal remains formerly analyzed are represented by empty squares.

The preservation of endogenous DNA in fossils is correlated with the amount, composition, and chemical preservation of amino acids (Poinar et al. 1996). We find that endogenous DNA can be amplified from Pleistocene remains when the amino acid content is more than 30,000 parts per million (ppm), the ratio of glycine to aspartic acid between two and ten, and the aspartic acid racemization (i.e., the stereoisomeric D/L ratio) less than 0.10 (Poinar et al. 1996; Krings et al. 1997, 2000; Schmitz et al. 2002; data not shown). We analyzed the amino acid preservation of 24 Neandertal and 40 early modern human fossils (Table S1). Several important Neandertal fossils, such as La Ferrassie and Krapina, as well as important modern human fossils, such as Veternica, proved to be too poorly preserved to be likely to allow DNA retrieval. Thus, further destructive sampling of these specimens was not considered justified. However, four Neandertal and five early modern human fossils fulfilled the above criteria for amino acid preservation and were thus expected to contain endogenous DNA (Figure 1; Table 1). These samples were geographically well distributed across Europe (Figure 2) and included remains whose morphology is typical of Neandertals (e.g., La Chapelle-aux-Saints) and of modern humans (La Madeleine, Cro-Magnon). They also included samples that have sometimes been considered “transitional” between Neandertals and modern humans, based on their morphological features: Vindija (Smith 1984) and Mladecˇ (Frayer 1986, Frayer 1992; Wolpoff 1999).

**Table 1:**
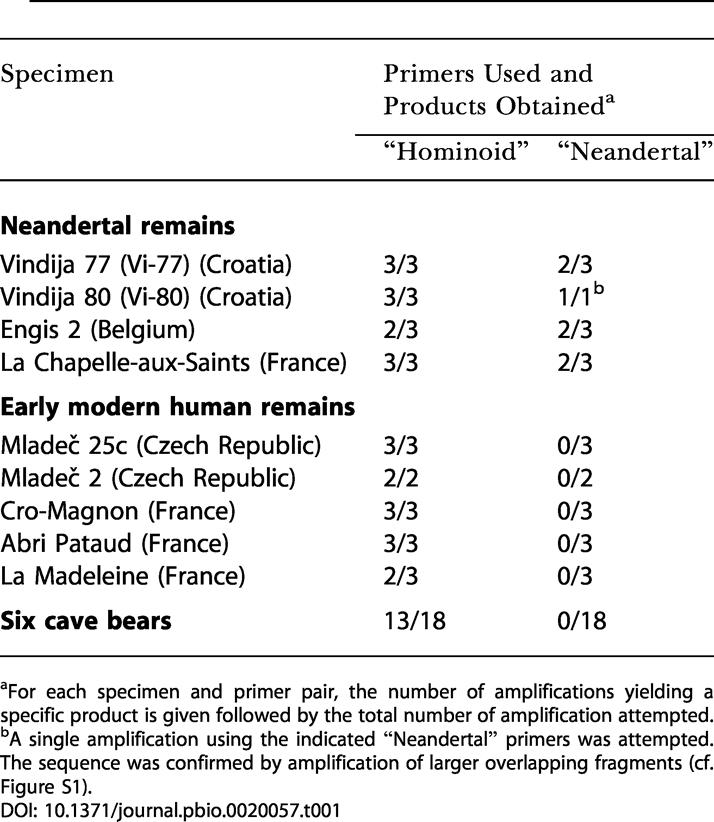
DNA Retrieved from Late Pleistocene Fossils in This Study

^a^For each specimen and primer pair, the number of amplifications yielding a specific product is given followed by the total number of amplification attempted

^b^A single amplification using the indicated “Neandertal” primers was attempted. The sequence was confirmed by amplification of larger overlapping fragments (cf. Figure S1)

If low amounts of DNA are preserved in a specimen, some extracts will fail to contain DNA molecules by chance (Hofreiter et al. 2001a). Therefore, except in the case of Mladecˇ 2, in which the amount of material available permitted only two extractions, we extracted each of the four Neandertal and the five early modern human samples three times. For each extraction, amplifications were performed using two primer pairs: (i) “hominoid primers” that amplify homologous mtDNA sequences from the previously determined Neandertals and contemporary modern humans, as well as African great apes; (ii) “Neandertal primers” that, under the conditions used, amplify only Neandertal mtDNAs even in the presence of a large excess of modern human DNA (Krings et al. 2000; Schmitz et al. 2002). Since authentic ancient DNA is typically highly degraded, both primer pairs were designed to amplify short mtDNA fragments (72 and 31 bp, respectively, excluding primers). In each of these fragments, two substitutions allow the discrimination of previously determined Neandertal mtDNA sequences from contemporary modern human sequences. The sensitivity of both primer pairs is similar, as shown by the fact that they are both able to amplify single template molecules as judged from nucleotide misincorporation patterns (Hofreiter et al. 2001a). In order to determine the nature of the DNA sequences amplified, each amplification product was cloned and approximately 30 clones were sequenced for each “hominoid product” and ten clones for each “Neandertal product.”

When amplified with the hominoid primers, all Neandertal and all early modern human remains yielded modern human DNA sequences (see Table 1). In addition, five cave bear teeth from Vindija, Croatia, and one from Gamssulzen, Austria, extracted in parallel with the hominid samples, all yielded human sequences. This confirms previous results in showing that most, if not all, ancient remains yield human DNA sequences when amplification conditions that allow single DNA molecules to be detected are used (Hofreiter et al. 2001b). For three Neandertal and all five modern human remains, several different mtDNA sequences were retrieved from individual extractions, and in the case of one Neandertal and one modern human, at least two of the sequences were also found in an independent extraction from the same specimen. Additionally, one of the cave bear teeth yielded a human sequence found in two independent extracts. Thus, the fact that a DNA sequence is found in two independent extracts is a necessary, but not sufficient, criterion of authenticity when human remains are analyzed. This implies that in the absence of further technical improvements, it is impossible to produce undisputable human mtDNA sequences from ancient human remains. In addition to DNA sequences identical to those previously amplified from present-day humans, the Neandertal bones Vi-77 and Vi-80 from Vindija yielded four out of 89 and 73 out of 85 mtDNA sequences, respectively, that were identical to previously determined Neandertal sequences. Thus, these two specimens contain a proportion of Neandertal-like mtDNA sequences (i.e., sequences that carry two substitutions that differentiate Neandertal mtDNA sequences from modern human mtDNA sequences as described above) that is high enough to detect using primers that amplify also modern human DNA.

**Figure 3:**
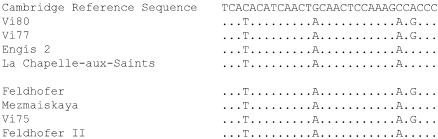
Sequences Obtained from the Neandertal Remains Using the “Neandertal Primers” Dots indicate identity to the human reference sequence (Anderson et al. 1981) given above. The four upper DNA sequences were determined in this study. Previously determined DNA sequences are shown below.

When amplified with Neandertal-specific primers, Neandertal-like mtDNA sequences were amplified from two independent extractions from all Neandertal fossils (see Table 1; Figure 3). For one of these, Vi-80 from Vindija, DNA preservation was sufficient to allow the retrieval of longer fragments and thus the reconstruction of 357 bp of the hypervariable region I (see Supporting Information section; Figure S1). This mtDNA sequence was identical to that retrieved from another bone from the same locality (Vi-75; Krings et al. 2000). In contrast to the Neandertal remains, none of the early modern human extracts yielded any amplification products with the Neandertal primers, although these remains are similar in chemical preservation to the Neandertal remains (see Figure 1).

Thus, all Neandertal remains analyzed yielded mtDNA sequences that are not found in the human mtDNA gene pool today but are similar to those found in four previously published Neandertals (Krings et al. 1997, Krings et al. 2000; Ovchinnikov et al. 2000; Schmitz et al. 2002) (see Figure 3). This is compatible with results suggesting that the extent of Neandertal mtDNA diversity was similar to that of current humans and lower than that of the great apes (Krings et al. 2000; Schmitz et al. 2002). It is noteworthy that this result is not an artifact created by discarding “modern-like” mtDNA sequences amplified from Neandertals (Trinkaus 2001), since all Neandertal remains with good biomolecular preservation yield “Neandertal-like” mtDNA sequence. Furthermore, none of the five early modern humans yields “Neandertal-like” mtDNA sequences in spite of the fact that these remains are as well preserved in terms of amino acids as the Neandertal remains. Thus, we fail to detect any evidence of mtDNA gene flow from Neandertals to early modern humans or from early modern humans to Neandertals.

**Figure 4:**
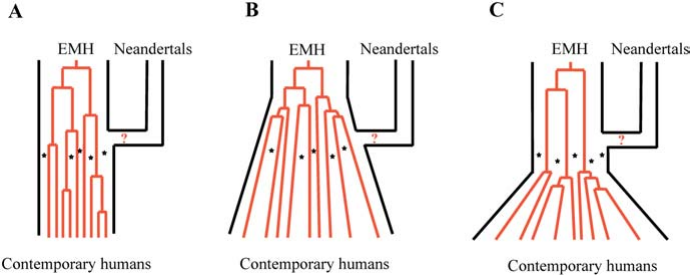
Schematic Model of Putative Contribution of Neandertal mtDNA to the Gene Pool of Modern Humans (A) Under the assumption of a constant effective population size of 10,000 for modern humans, contemporary mtDNAs trace back to approximately five mtDNA lineages 25,000 years ago. The modern human fossils represent five additional samples from around the time of putative admixture (stars). The contemporary and early modern human (EMH) samples reject a Neandertal contribution of 25% or more to modern humans about 30,000 years ago (*p* ≤ 0.05). (B) Under the more realistic scenario of an expansion of the human population during and after the colonization of Europe, a smaller Neandertal contribution can be excluded because the number of ancestors of the current human gene pool was larger 30,000 years ago. However, the contribution that can be excluded would depend on when and how the expansion occurred. (C) Under the scenario that population size was constant before a putative merging with the Neandertal population and expanded only thereafter, the Neandertal contribution could have been larger, but similarly depends on how the expansion occurred.

However, a relevant question is what extent of gene flow between Neandertals and early modern humans the current data allow us to exclude. In this regard, it is of relevance that the five early modern humans analyzed lived much closer in time to the Neandertals than do contemporary individuals. The probability that mtDNA sequences potentially contributed to modern humans by Neandertals were lost by drift (Nordborg 1998) or swamped by continuous influx of modern human mtDNAs (Enflo et al. 2001) in the Neandertal gene pool is therefore much smaller than when contemporary humans are analyzed (e.g., Relethford 1999). In fact, the five early modern humans analyzed almost double the amount of information about the Upper Pleistocene mtDNA gene pool since, under a model of constant effective population size, all contemporary humans trace their mtDNA ancestors back to only four to seven mtDNA lineages 20,000 to 30,000 years ago (Figure 4A; Figure S2), while all other mtDNA sequences present in the gene pool at that time have been lost by random genetic drift. Since the probability is very low (*p* < 0.007) that one or more of the five early modern humans analyzed here are among these few ancestors of current humans, the five Upper Pleistocene individuals can be added to the ancestors of the current mtDNA gene pool to allow us to ask what extent of Neandertal mtDNA contribution to early modern humans can be statistically excluded using the coalescent. Under the model of a constant human effective population size (Tavare 1984; Nordborg 1998) of 10,000 over time (Figure 4A), any contribution of Neandertal mtDNA to modern humans 30,000 years ago larger than 25% can be excluded at the 5% level (Figure S3). A more realistic scenario may be that the spread of modern humans was accompanied by an increase in population size before and during their migration out of Africa and subsequent colonization of western Eurasia (see Figure 4B). In that case, the Neandertal contribution that can be excluded is smaller (i.e., less gene flow could have taken place), but that depends critically on when and how the expansion occurred. Finally, under the unlikely scenario that population size was constant during the migration out of Africa and colonization of Europe and expanded only after a putative merging with Neandertals, the Neandertal contribution could have been larger, but this also depends on the nature of the growth (see Figure 4C).

## Concluding Remarks

It is noteworthy that under the model of constant population size, about 50 early modern human remains would need to be studied to exclude a Neandertal mtDNA contribution of 10%. To exclude a 5% contribution, one would need to study more early modern human remains than have been discovered to date. Thus, definitive knowledge of the extent of a putative contribution of Neandertals to the modern human gene pool will not be possible, although extensive studies of variation in the current human gene pool may clarify this question (Wall 2000). It is, however, worthwhile to note that samples considered as anatomically “transitional” between modern humans and Neandertals, such as Vindija (Smith 1984; Wolpoff 1999) and Mladecˇ (Frayer 1986, Frayer 1992; Wolpoff 1999), analyzed here, fail to show any evidence of mtDNA admixture between the two groups. Thus, while it cannot be excluded that Neandertals contributed variants at some genetic loci to contemporary humans, no positive evidence of any such contribution has yet been detected.

## Materials and Methods

### 

#### Amino acid preservation

About 10 mg of bone were removed from each specimen and analyzed as in Schmitz et al. (2002) with minor modifications. In brief, proteins are hydrolyzed and amino acids labeled with *o*-phtaldialdehyde/*N*-acetyl-L-cysteine and analyzed by high performance liquid chromatography (Shimadzu, Kyoto, Japan) under conditions that separate the different amino acids as well as their stereoisomers. Eight amino acids are analyzed and their respective concentration measured: D- and L-alanine, glycine, D- and L-aspartic acid, serine, glutamic acid, valine, D- and L-leucine, and isoleucine.

#### DNA extraction and amplification

DNA extractions were performed in a laboratory dedicated to ancient DNA work. In this laboratory, positive air pressure is maintained with filtered air at all times, and all areas and equipment are treated with UV light when the laboratory is not used. A maximum of six bone or teeth samples were processed together with two blank extractions. Neandertal samples were always processed together with early modern human samples or cave bear samples. For each extraction, the samples were ground and between 30 mg and 120 mg of bone powder was extracted as in Krings et al. (1997). mtDNA sequences were amplified by polymerase chain reaction (PCR) using 5 μl of extract and 60 cycles. In addition, a minimum of four blank PCRs were performed together with each amplification from extracts. The “Neandertal-specific” amplification was carried out using the primers NL16230/NH16262 (Krings et al. 1997) and an annealing temperature of 60°C. We consider it highly unlikely that the Neandertal-specific mtDNA fragments represent contaminations from other Neandertals, given that none of the extracts of modern humans or cave bears processed in parallel with the Neandertal remains yielded such products. The “hominoid” amplification was performed with the primers L16022/H16095 (Krings et al. 1997) and an annealing temperature of 54°C. PCR products were cloned into Escherichia coli using the TOPO TA cloning kit (Invitrogen, Leek, The Netherlands), and ten or 30 clones of each amplification were sequenced on a ABI 3700 (Applied Biosystems, Foster City, California, United States).

####  Estimation of admixture

Given that previous analyses of mtDNA sequences have rejected a model of complete panmixia between Neandertals and early modern humans (Nordborg 1998), we focused on the estimation of the level of admixture between Neandertals and early modern humans that can be excluded. For this purpose, we considered a population of early modern humans that merged at *Tm* with a (genetically different) population of Neandertal individuals (see Figure 4) from which point the fused population was panmictic. The probability of picking *K* individuals by chance in the merged population that all carry a modern human mtDNA sequence is (1 − *c*)*^K^*, where *c* represents the Neandertal genetic contribution to the merged population. If none of *n* mtDNA sequences sampled in the merged population is Neandertal-like, we can exclude (at the 5% level) contributions that give a probability smaller than 0.05 of observing only modern human sequences, i.e., (1 − *c*)*^K^* < 0.05. The number of ancestors of *n* samples at the time *t* is represented by a probability distribution, A*n*(*t*). Thus, the probability of observing only one kind of sequences in *n* samples becomes:



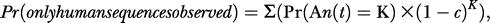



where *K* vary from 1 to *n*. For a population of constant size over time, Pr(A*n*(*t*) = *K*) has been derived in Tavare (1984). We estimated the number of ancestors of *n* samples at time *t* as the expected value of A*n*(*t*), E(A*n*(*t*)), according to this model and calculate the probability of observing only human sequences for different values of *c*.

## Supporting Information

### Determination of the mtDNA Sequence of Vi-80 from Vindija, Croatia

The entire hypervariable region I sequence was determined from this specimen using amplifications and clones given in Figure S1. Its sequence is identical to the sequence previously determined from individual Vi-75 from Vindija (Krings et al. 2000). We could exclude cross-contamination from the old extract to this bone because different primers were used and some of the fragments of mtDNA amplified from Vi-80 were longer than those used to determine the sequence of Vi-75. Morphological analyses do not exclude that these two fragmentary bones (Vi-75 and Vi-80) may come from a single individual. Carbon-14 accelerator mass spectrometry dating, conducted in the Ångstrom Laboratory (Uppsala University, Sweden), yielded a date for Vi-80 of 38,310 ± 2,130 BP (before present). Since Vi-75 has been previously dated to over 42,000 BP (Krings et al. 2000), the possibility exists that the dates overlap since 42,000 BP is within two standard deviations of the Vi-80 date. Therefore, the bone labeled Vi-80 that yields the new mtDNA sequence could either be (i) a fragment of the same skeleton (individual) that was already successfully extracted, (ii) a bone from another individual maternally related to the first individual amplified, or (iii) another unrelated individual having by chance the same mtDNA sequence, which is not unlikely given the apparently low mtDNA diversity of Neandertals (Krings et al. 2000; Schmitz et al. 2002).

Figure S1 The DNA Sequences of the Clones Used to Reconstruct the Sequence of the Mitochondrial Hypervariable Region I from the Bone Vi-80 (30 KB PDF).

Figure S2 Expected Number of Ancestors E(A*n*(*t*)) of *n* Individuals under a Model of Constant Population Size of Ne = 10,000 The number of ancestors of *n* individuals (x axis) is estimated at 20,000, 25,000, and 30,000 years ago. For example, 150 humans living today have approximately seven ancestors 20,000 years ago.
(56 KB PDF).

Figure S3 Probability of Different Levels of Admixture Probability of observing only modern human mtDNA sequences in both five early human remains and the current mtDNA gene pool given different proportion of Neandertal contribution *c* (x axis) under a model of constant population size (see text; Materials and Methods). For example, the probability of observing only human mtDNA sequences given a Neandertal contribution of 25% or more is smaller than 0.05 (dotted line).
(42 KB PDF).

Table S1 Results of the Amino Acid Analyses of 40 Human and 24 Neandertal Remains The bones were analyzed by high performance liquid chromatography for their amino acid content (see Materials and Methods). The extent of racemization of aspartic acid (D-/L-Asp), the ratio of glycine to aspartic acid (Gly/Asp), and the total amount of the eight amino acid analyzed (ppm) are given for each specimen. Zero indicates values below detection level. The five human and four Neandertal specimens from which DNA extraction were performed are displayed in green.
(54 KB PDF).

## Abbreviations

BP: before present
mtDNA: mitochondrial DNA
PCR: polymerase chain reaction
ppm: parts per million
