# Supplementary material for: No Evidence of Neandertal mtDNA Contribution to Early Modern Humans
Source: PLoS Biol. 2004 Mar 16;2(3):e57. doi: 10.1371/journal.pbio.0020057 (PMC368159; doi:10.1371/journal.pbio.0020057)
Supplement: Figure S1 — (30 KB PDF). [file pbio.0020057.sg001.pdf]

```
ATTCTAATTTAAACTATTCTCTGTTCTTTCATGGGGAAGCAGATTTGGGTACCACCCAAGTATTGACTCACCCATCAACAACCGCTATGTATTTCGTACATTACTGCCAGCCACCATGAATATTGTACGGTACCATAAATACTTGACCACCTGTAGTACATAAAAACCCAATCCACATCAAAACCCCCTCCCCATGCTTACAAGCAAGTACAGCAATCAACCCTCAACTATCACACATCAACTGCAACTCCAAAGCCACCCCT-CACCCACTAGGATACCAACAAACCTACCCACCCTTAACAGTACATAGTACAT-AAAGCCATTT-ACCGTACATAGCACATTACAG-TCAAATCCCTTCTCGTCCCCATGGATGACCCCCCTCAGATAGGGGTCCCTTG
ATTCTAATTTAAACTATTCTCTGTTCTTTCATGGGGAAGCAGATTTGGGTACCACCCAAGTATTGACTCACCCATCAACAACCGCTATGTATTTCGTACATTACTGCCAGCCAC
ATTCTAATTTAAACTATTCTCT..............G........................................G................CGTACATTACTGCCAGCCAC
L16.022  #2046        ..............G........................................G................         H16.095  #3
                      ..............G........................................G................
                      ..............G........................................G................
                      ..............G........................................G................
                      ..............G........................................G................
                      ..............G........................................G................
                      ..............G.................................TT.....G................
                      ..............G........................................G................
                      .....C........G...........-...-........................G......-.........
                      ..............G........................................G................
                      ..............G....................T...................G................
                      ..............G...............................................T.........
                      ..............G........................................G................
                      ..............G........................................G................
ATTCTAATTTAAACTATTCTCT..............G........................................G................CGTACATTACTGCCAGCCAC
L16.022  #2046        ..............G........................................G................         H16.095  #3
                      ..............G........................................G................
                      ..............G..................T..........T...T.T....G................
                      ..............G........................................G................
                      ..............G........................................G................
                      ..............G........................................G................
                      ..............G........................................G................
                      ..............G........................................G................
                      ..............G........................................G................
                      ..............G........................................G................
                      ..............G........................................G................
                      ..............G........................................G................
                      ..............G.................TT.....................G................
                      ..............G.................TT.....................G................
                      ..............G.................TT.....................G................
                      ..............G........................................G................
                      ..............G........................................G................
                      ..............G........................................G................

GAAGCAGATTTGGGTACCACCCAAGTATTGACTCACCCATCAACAACCGCTATGTATTTCGTACATTACTGCCAGCCACCATGAATATTGTACGGTACCATAAATACTTGACCACCTGCAGTACAT
GAAGCAGATTTGGGTACCAC......................G..................................................A.........TTACTTGACTACCTGCAGTACAT
L16.055  #1         ......................G..................................................A.........        NH16.139  #2091
                    ......................G..................................................A.........
GAAGCAGATTTGGGTACCAC......................G..................................................A.........TTACTTGACTACCTGCAGTACAT
L16.055  #1         ......................G..................................................A.........        NH16.139  #2091
                    ......................G..................................................A.........
                    ......................G..................................................A.........
                    ......................G..................................................A.........

CATTACTGCCAGCCACCATGAATATTGTACGGTACCATAAATACTTGACCACCTGTAGTACATAAAAACCCAATCCACATCAAAACCCCCTCCCCATG-CTTACAAGCAAGTACAGCAATCAACCCTCAACTATCACACATCAACTGCAACTCCAAAGCCACCCCT-CACCCACTAGGAT
CATTACTGCCAGCCACCATGAATA......A.........T........T.....C..............T.............CC....C.......G............C.M...........T.M....G...A.........A..ACTCCAAAGCCACCCCT-CACCCACTAGGAT
L16.122  #1989          ......A.........T........T.....C..............T.............CC....C.......G............C.............T......G...W.........A..               H16.247   #2769
                        .........................T.....C.............................................................................................
                        .....................................................................................................T......G...T.........A..
CATTACTGCCAGCCACCATGAATA.....TA.........T........T.....C..............T.............CC....C....................C.............T......G...T.........A..ACTCCAAAGCCACCCCT-CACCCACTAGGAT
L16.122  #1989          .....TA.........T........T.....C..............T......................T.......................................................               H16.247   #2769
                        .............C...........................M...........................T......................................G...T.........A..
                        .....TA.........T........T.....C..............T.............CC....C..-.................C.............T......G...T.........A..
                        .....TA.........T........T.....C..............T.............CC....C..-.................C.............T......G...T.........A..
                        ....N.A.........T........T.....C..............T.............CC....C..-.................C.............T......G...T.........AT.
                        .....TA.........T........T.....C..............T.....N.......CC....C....................C....A................................
                        .....TA.........T........T.....C.....................................T.......................................................
                        .....TA.........T........T.....C.....................................T.......................................................
                        .............C.......................................................T.................C.............T......G...T.........A..

GTACAGCAATCAACCCTCAACTATCACACATCAACTGCAACTCCAAAGCCACCCCT-CACCCACTAGGATACCAACAAACCTAC
GCACAGCAATCAACCTTCAACTG...T.........A...........A.G...TTACACCCACTAGGATATCAACAAATCTAC
NL16.230  #2480        ...T.........A...........A.G...                    NH16.262  #2481
                       ...T.........A...........A.G...
                       ...T.........A...........A.G...
                       ...T.........A...........A.G...
                       ...T.........A...........A.G...
                       ...T.........A...........A.G...
                       ...T....T....A.....GC....A.G...

GTACAGCAATCAACCCTCAACTATCACACATCAACTGCAACTCCAAAGCCACCCCT-CACCCACTAGGATACCAACAAACCTACCCACCCTTAACAG-TACATAGTACATAAAGCCATTTACCGTACATAGCACATTACAGTCAAATCCCTTCTCGTCCCCATGGATGACCCCCCTCAGATAGGGGTCCCTTG
GCACAGCAATCAACCTTCAACTG...T.........A...........A.G...T.A..............T....................G............C........T.........................................C.TTT.............CCTCAGATAGGGGTCCCTTG
NL16.230  #2480        ...T.........A...........A.G...T.A..............T....................G............C........T.........................................C.TTT.............       H16378  #324
                       ...T.........A...........A.G...T.A..............T....................G............C........T.........................................C.TTT.............
                       ...T.........A...........A.G...T.A..............T....................G............C........T.........................................C.TTT.............
                       ...T.........A...........A.G...T.A..............T....................G............C........T.........................................C.TTT.............
                       ...T.........A...........A.G...T.A..............T....................G............C........T.........................................C.TTT.............
                       ...T.........A...........A.G.T.T.A..............T........T...........G............C........T.........................................C.................
                       ...T.........A...........A.G.T.T.A..............T........T...........G............C........T.........................................C............M....
                       ...T.........A...........A.G.T.T.A..............T........T...........G............C........T.........................................C.................
                       ...T.........A...........A.G.T.T.A..............T........T...........G............C........T.........................................C.................
                       ...T.........A...........A.G.T.T.A..............T........T...........G............C........T.........................................C.................
                       ...T.........A...........A.G.T.T.A..............T........T...........G............C........T.........................................C.................
                       ...T.........A...........A.G.T.T.A..............T........T...........G............C........T.........................................C.................
                       ...T.........A...........A.G.T.T.A..............T........T...........G............C........T.........................................C.................
                       ...T.........A...........A.G.T.T.A..............T........T...........G............C........T.........................................C.................

GTACAGCAATCAACCCTCAACTATCACACATCAACTGCAACTCCAAAGCCACCCCT-CACCCACTAGGATACCAACAAACCTACCCACCCTTAACAG-TACATAGTACATAAAGCCATTTACCGTACATAG
GCACAGCAATCAACCTTCAACTG...T.........A...........A.G...T.A..............T....................G............CACATAAAGTCATTTACCGTACATAG
NL16.230  #2480        ...T.........A...........A.G...T.A..............T....................G............           NH 16.311 #311
                       ...T.........A...........A.G...T.A..............T....................G............

AACAAACCTACCCACCCTTAACAG-TACATAGTACATAAAGCCATTTACCGTACATAGCACATTACAGTCAAATCCCTTCTCGTCCCCATGGATGACCCCCCTCAGATAGGGGTCCCTTG
AACAAACCTACCCACCCTTG............C........T.........................................C.................CCTCAGATAGGGGTCCCTTG
NL 16299  #2853     ............C........T...........................................T.T.............       H16378  #324
                    ............C........T.........................................C.................
                    ............C........T.........................................C.................
                    ............C........T.........................................C.................

ATTCTAATTTAAACTATTCTCTGTTCTTTCATGGGGAAGCAGATTTGGGTACCACCCAAGTATTGACTCACCCATCAACAACCGCTATGTATTTCGTACATTACTGCCAGCCACCATGAATATTGTACGGTACCATAAATACTTGACCACCTGTAGTACATAAAAACCCAATCCACATCAAAACCCCCTCCCC-ATGCTTACAAGCAAGTACAGCAATCAACCCTCAACTATCACACATCAACTGCAACTCCAAAGCCACCCCT-CACCCACTAGGATACCAACAAACCTACCCACCCTTAACAG-TACATAGTACATAAAGCCATTTACCGTACATAGCACATTACAGTCAAATCCCTTCTCGTCCCCA-TGG-ATGACCCCCCTCAGATAGGGGTCCCTTG
Feldhofer II          ..............G........................................G..................................................A.........T........T.....C..............T.............CC....C....................C.............T......G...T.........A...........A.....T.A..............T....................G............C........T.........................................C..................
Feldhofer    (1997)   ..............G........................................G..............C.............TT..TT................A.........T........T.....C..............T..............C....C....................C.............T......G...T.........A...........A.G...T.A..............T....................G............C........T.........................................C..................
Vindija75    (2000)   ..............G........................................G..................................................A.........T........T.....C..............T.............CC....C....................C.............T......G...T.........A...........A.G...T.A..............T....................G............C........T.........................................C..................
Mezmaiskaya  (2000)   ...............................................................C..........................................A.........T........T.......A............T.............CC....C....................C.............T......G...T.........A...........A.....T.A..............T....................G............C........T.......................T.................C..................
```
