# Supplementary figures and images for: No Evidence of Neandertal mtDNA Contribution to Early Modern Humans
Source: PLoS Biol. 2004 Mar 16;2(3):e57. doi: 10.1371/journal.pbio.0020057 (PMC368159; doi:10.1371/journal.pbio.0020057)

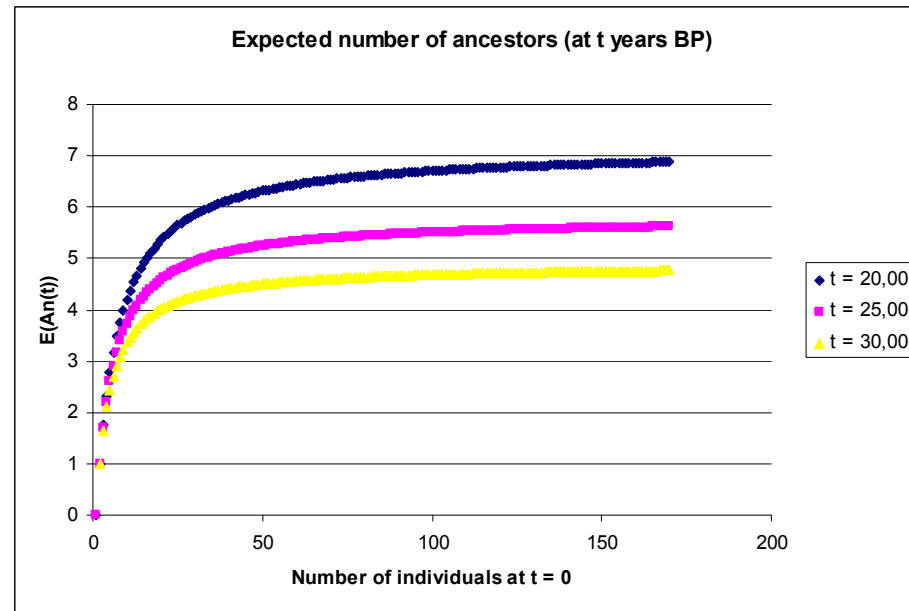

Suppl. Figure S2

Supplement: Figure S2 — The number of ancestors of n individuals (x axis) is estimated at 20,000, 25,000, and 30,000 years ago. For example, 150 humans living today have approximately seven ancestors 20,000 years ago. (56 KB PDF). [file pbio.0020057.sg002.pdf]

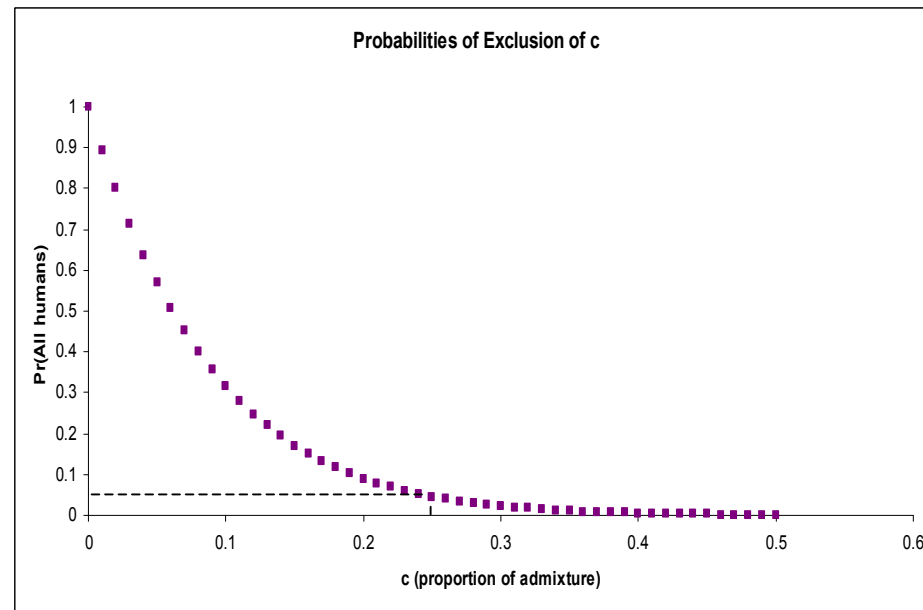

Suppl. Figure S3

Supplement: Figure S3 — Probability of observing only modern human mtDNA sequences in both five early human remains and the current mtDNA gene pool given different proportion of Neandertal contribution c (x axis) under a model of constant population size (see text; Materials and Methods). For example, the probability of observing only human mtDNA sequences given a Neandertal contribution of 25% or more is smaller than 0.05 (dotted line). (42 KB PDF). [file pbio.0020057.sg003.pdf]
