# Supplementary material for: No Evidence of Neandertal mtDNA Contribution to Early Modern Humans
Source: PLoS Biol. 2004 Mar 16;2(3):e57. doi: 10.1371/journal.pbio.0020057 (PMC368159; doi:10.1371/journal.pbio.0020057)
Supplement: Table S1 — The bones were analyzed by high performance liquid chromatography for their amino acid content (see Materials and Methods). The extent of racemization of aspartic acid (D-/L-Asp), the ratio of glycine to aspartic acid (Gly/Asp), and the total amount of the eight amino acid analyzed (ppm) are given for each specimen. Zero indicates values below detection level. The five human and four Neandertal specimens from which DNA extraction were performed are displayed in green. (54 KB PDF). [file pbio.0020057.st001.pdf]

Supplementary Table 1. Amino acid analyses of 64 hominid remains

Specimen/Provenience	Taxon	D/L Asp	Gly/Asp	ppm
Abri Pataud, France	Modern Humans	0.086	3.18	14380
Abri Pataud, France	Modern Humans	0	3.87	24817
Abri Pataud, France	Modern Humans	0	0	0
Abri Pataud, France	Modern Humans	0.068	4.61	24743
Abri Pataud, France	Modern Humans	0	3.77	17582
Abri Pataud, France	Modern Humans	0.062	7.37	56119
Cro Magnon, France	Modern Humans	0	6.98	6438
Cro Magnon, France	Modern Humans	0.011	2.11	2287
Cro Magnon, France	Modern Humans	0	1.9	1784
Cro Magnon, France	Modern Humans	0.095	1.7	2060
Cro Magnon, France	Modern Humans	0.117	2.97	13532
Cro Magnon, France	Modern Humans	0.081	7.59	56684
La Madeleine, France	Modern Humans	0	5.02	14384
La Madeleine, France	Modern Humans	0	5.34	18880
La Madeleine, France	Modern Humans	0	0	0
La Madeleine, France	Modern Humans	0.096	1.17	4985
La Madeleine, France	Modern Humans	0.095	1.75	7688
La Madeleine, France	Modern Humans	0.086	0.78	5256
La Madeleine, France	Modern Humans	0.091	7.21	29901
Mladec 2, Czech Republic	Modern Humans	0.080	8.25	87436
Mladec 21, Czech Republic	Modern Humans	0.330	13.71	4895
Mladec 25a, Czech Republic	Modern Humans	0.003	8.22	14565
Mladec 25c, Czech Republic	Modern Humans	0.009	9.06	53718
Mladec 26, Czech Republic	Modern Humans	0	9.89	3253
Mladec 27, Czech Republic	Modern Humans	0	113.48	24156
Mladec 27, Czech Republic	Modern Humans	0	11.81	3883
Mladec 28, Czech Republic	Modern Humans	0	8.53	3343
Mladec 28, Czech Republic	Modern Humans	0	9.69	2151
Mladec 33, Czech Republic	Modern Humans	0	11.37	4001
Mladec 8, Czech Republic	Modern Humans	0.133	5.21	3488
Mladec child, Czech Republic	Modern Humans	0	115.01	9308
Sandalja, Croatia	Modern Humans	0.115	0.97	10848
Sandalja, Croatia	Modern Humans	0.12	0.95	22256
Sandalja, Croatia	Modern Humans	0.111	1.19	10950
Veternica, Croatia	Modern Humans	0.089	3.7	9429
Veternica, Croatia	Modern Humans	0.092	1	11886
Veternica, Croatia	Modern Humans	0.066	1.09	12218
Veternica, Croatia	Modern Humans	0.082	6.7	23509
Veternica, Croatia	Modern Humans	0.076	1.37	22340
Veternica, Croatia	Modern Humans	0.07	1.11	13161
Monte Circeo, Italia	Neandertal	0.244	4.57	371
Amud, Israel	Neandertal	0.171	1.79	164
Dederiyeh, Syria	Neandertal	0.000	4.48	200
Dederiyeh, Syria	Neandertal	0.000	0	0
El Sidron, Spain	Neandertal	0.053	9.92	9924
Engis 2, Belgium	Neandertal	0.086	7.55	31561
Krapina, Croatia	Neandertal	0.223	3.77	7044
Krapina, Croatia	Neandertal	0	0	0
Krapina, Croatia	Neandertal	0	0	0
La Ferassie 2, France	Neandertal	0.033	9.80	1419
La Ferassie 1, France	Neandertal	0.251	7.12	40353

La Ferassie 1, France	Neandertal	0.296	2.06	85510
La Ferassie 1, France	Neandertal	0.263	3.13	30150
La Ferassie 1, France	Neandertal	0.165	6.07	36405
La Ferassie 1, France	Neandertal	0.141	4.28	42145
La-Chapelle-aux-Saints, France	Neandertal	0	7.49	2190
La-Chapelle-aux-Saints, France	Neandertal	0.068	4.89	19650
La-Chapelle-aux-Saints, France	Neandertal	0.065	7.97	55128
LaQuina, France	Neandertal	0	0	0
LaQuina, France	Neandertal	0.081	1	2931
LaQuina, France	Neandertal	0.225	0	194
LaQuina, France	Neandertal	0	0	0
Vindija Vi-80, Croatia	Neandertal	0.074	5.95	45212
Vindija Vi-77, Croatia	Neandertal	0.071	3.01	74596
